# Epidemiology of potential drug- drug interactions in hospitalized patients with type 2 diabetes mellitus in China: a retrospective study

**DOI:** 10.3389/fendo.2024.1387242

**Published:** 2024-06-25

**Authors:** Weifang Ren, Yujuan Liu, Huaqiao Jiang, Xiaoqun Lv, Ning Zhang

**Affiliations:** Department of Pharmacy, Jinshan Hospital, Fudan University, Shanghai, China

**Keywords:** drug-drug interactions, herb-drug interactions, type 2 diabetes mellitus, hospitalized patients, discharge

## Abstract

**Background:**

Combination therapy was associated with an increased risk of drug- drug interactions (DDIs) in patients with type 2 diabetes mellitus (T2DM). The present study aimed to investigate the epidemiology of potential DDIs (pDDIs), including potential chemical drug-drug interactions (pCDIs) and potential herb-drug interactions (pHDIs), and classify the influencing factors of pDDIs in these patients.

**Methods:**

A retrospective study of the epidemiology of pDDIs among T2DM hospitalized patients older than 18 years and treated with at least two drugs during hospitalization was conducted over a 12-month period in 2019. PDDIs were identified with C (monitor therapy), D (consider therapy modification), and X (avoid combination) risk ratings. Binary logistic regression was used to analyze the risk factors of pDDIs.

**Results:**

A total of 6796 pDDIs were identified from 737 T2DM hospitalized patients during hospitalization, with 0.87% classified as X risk rating, 13.39% as D risk rating. Additionally, 1753 pDDIs were identified after discharge, with 0.11% as X and 25.73% as D risk rating. The drug-drug association networks showed that the majority of pCDIs were associated with cardiovascular system drugs. Chlorphenamine-potassium chloride and danshen-warfarin were the most prevalent interacting pairs of pCDIs and pHDIs with X rating during hospitalization. Multivariate analysis indicated that the likelihood of developing over 4 pDDIs was significantly higher among T2DM patients who had received over 8 medications. The presence of pDDIs after discharge was strongly associated with the complications of T2DM and the number of discharge medications.

**Conclusions:**

T2DM patients were frequently exposed to pDDIs, including pCDIs and pHDIs, both during hospitalization and after discharge. Multi-drug combination was the primary risk factor for pDDIs. Strategies such as enhancing the monitoring and warning for pDDIs, increasing clinical pharmacological experience, as well as developing universally applicable clinical guidelines for pDDIs may be beneficial in reducing the incidence of potentially harmful drug-combinations.

## Introduction

The benefits of medication therapy for patients are evident in preventing and treating various diseases, as well as improving or maintaining the quality of life (1). However, individuals with multimorbidity often experience poor functional status and health outcomes, and are frequently treated by multiple healthcare specialists, resulting in frequent hospital visits, polypharmacy, and a substantial treatment burden (2–4). Polypharmacy is associated with a significantly increased risk of drug-drug interactions (DDIs) and adverse drug events (ADEs) among patients with multiple comorbidities (5, 6).

As one of the preventable drug-related problems, DDIs occur when a patient is simultaneously exposed to two or more medications that are known to interact. These interactions might have positive effects by enhancing effectiveness or negative effects by contributing to ADEs and toxicity (7). In addition, DDIs might reduce therapeutic efficacy by exerting inhibitory or inductive effects on cytochrome P450 (8). The risk of ADEs arises from DDIs when multiple medications are taken (9). Therefore, it is crucial to evaluate the possibility and severity of DDIs, as well as to identify the factors that influence them.

T2DM patients often have a high prevalence of chronic comorbidities and frequently experience polypharmacy (10, 11), putting them at a high risk for DDIs especially during hospitalization. Previous studies have retrospectively determined that the prevalence of pDDIs in T2DM patients varied from 10% to 81% in different studies (12–14). However, few studies have considered the influence of herbs or Chinese patent medicines, which are composed of several Chinese medicinal herbs with high consumption in patients with T2DM. Hence, we aimed to retrospectively investigate the epidemiology of pDDIs involving both chemical drugs and herbs or Chinese patent medicines, and to classify the factors influencing pDDIs (pCDIs+pHDIs) in T2DM hospitalized patients, both during hospitalization and after discharge.

## Materials and methods

### Study design and data collection

This retrospective study was conducted to determine the prevalence of pDDIs (pCDIs+pHDIs), and explore the contributing factors of pDDIs among T2DM inpatients in the endocrine department of Jinshan Hospital, Fudan University, a large-scale general university hospital in the Jinshan District of Shanghai, China. From January 1^st^ to December 31^st^ in 2019, a total of 737 T2DM inpatients older than 18 years who were treated with at least two drugs during hospitalization were included in the study. Medications for topical treatment, the skin, and menstruum were excluded. Chinese patent drugs composed of several Chinese medicinal herbs were considered individually in the analysis. Patient information, including age, sex, diagnosis, duration of hospitalization, and medication use during hospitalization and after discharge, was obtained from medical records, medication orders, and discharge summaries.

PCDIs and pHDIs were identified using Lexi-Interact in UpToDate, Stockley’s Drug Interactions, and Medicine Specification in order of priority. All pDDIs (pCDIs+pHDIs) during hospitalization and after discharge were recorded and statistically analyzed, including C (monitor therapy), D (consider therapy modification), and X (avoid combination) risk ratings. PDDIs (pCDIs+pHDIs) with B (no action needed) and A (no known interaction) risk ratings were considered to have no clinically significant interactions and were consequently excluded.

### Data analysis

All data were coded and analyzed using SPSS 20.0. Descriptive statistics were presented as frequencies, percentages, mean and standard deviation (SD). Univariate and multivariate analyses were performed to analyze the risk factors associated with the occurrence of pDDIs (pCDIs+pHDIs) as a binary outcome. Independent variables (p-value<0.10) in the univariate analysis were included in the multivariate analysis by means of binary logistic regression. A p-value<0.05 was considered statistically significant. Network diagrams were generated using Cytoscape v3.7.2. In the network diagram, a node represented a drug.

## Results

### Patients’ characteristics

A total of 737 T2DM inpatients were in accordance with the inclusive criteria, including 411 (55.80%) males and 326 (44.20%) females. The demographic and clinical characteristics of the study population were summarized in **Table 1**. The mean age of the patients was 60 ± 15 years, with the range between 20 and 91. The most common comorbid conditions were hypertension (422 patients, 57.30%), hyperlipidemia (265 patients, 36.00%) and infection (99 patients, 13.40%). Among the patients, 57.67% were prescribed 8–13 drugs, while 27.95% more than 13 drugs and 14.38% 2–7 drugs. The average number of medications was 11.89 (range from 2 to 42) during hospitalization and 5.83 (range from 0 to 32) after discharge.

**Table 1:** Demographic and clinical characteristics of the study population and the total number of pDDIs during hospitalization and after discharge (n=737).

Characteristics	No. of patients, n (%)	No. of pDDIs (pCDIs+ pHDIs) , n (%)	
		During hospitalization	After discharge
Gender			
Male	411 (55.80)	3547 (52.19)	925 (52.80)
Female	326 (44.20)	3249 (47.81)	827 (47.20)
Age, (years)			
18-39	81 (11.00)	635 (9.34)	120 (6.85)
40-64	354 (48.00)	3055 (44.96)	772 (44.06)
≥65	302 (41.00)	3106 (45.70)	860 (49.09)
BMI			
Underweight (<18.5 kg/m^2^)	13 (1.80)	108 (1.59)	29 (1.65)
Normal (18.5-24.99 kg/m^2^)	328 (44.50)	2669 (39.27)	653 (37.21)
Preobese (25-29.99 kg/m^2^)	312 (42.30)	3024 (44.50)	818 (46.69)
Obese (≥30 kg/m^2^)	84 (11.40)	995 (14.64)	253 (14.44)
Length of hospitalization, (day)			
1-10	398 (54.00)	3373 (49.63)	943 (53.82)
>10	339 (46.00)	3423 (50.37)	809 (46.18)
History of T2DM, (years)			
0-10	529 (71.78)	4582 (67.42)	1127 (64.33)
>10	208 (28.22)	2214 (32.58)	625 (35.67)
Alcohol use history			
No	690 (93.60)	6418 (94.44)	1645 (93.89)
Yes	47 (6.40)	378 (5.56)	107 (6.11)
Tobacco use history			
No	656 (89.00)	6097 (89.71)	1570 (89.61)
Yes	81 (11.00)	699 (10.29)	182 (10.39)
Family history of T2DM			
No	525 (71.20)	4937 (72.65)	1271 (72.55)
Yes	212 (28.80)	1859 (27.35)	481 (27.45)
Complications of T2DM			
No	149 (20.20)	1144 (16.83)	237 (13.53)
Yes	588 (79.80)	5652 (83.17)	1515 (86.47)
Comorbid conditions			
Hypertension	422 (57.30)	4327 (63.67)	1185 (67.64)
Hyperlipidemia	265 (36.00)	2465 (36.27)	586 (33.45)
Coronary heart disease	84 (11.40)	1166 (17.16)	420 (23.97)
Chronic gastritis	30 (4.10)	268 (3.94)	84 (4.79)
Osteoporosis	55 (7.50)	599 (8.81)	159 (9.07)
Infection	99 (13.40)	1020 (15.01)	242 (13.81)
Number of complications			
0-2	181 (24.56)	1318 (19.39)	311 (17.75)
≥3	556 (75.44)	5478 (80.61)	1441 (82.25)
Medications during hospitalization			
2-7	106 (14.38)	493 (7.25)	–
8-13	425 (57.67)	3353 (49.34)	–
>13	206 (27.95)	2950 (43.41)	–
Discharge medications			
0-5	374 (50.75)	–	481 (27.45)
≥6	363 (49.25)	–	1271 (72.55)
		Mean±SD	
Number of mediations per patient in hospital		11.89±4.96	
Number of mediations per patient after discharge		5.83±2.99	
Number of pDDIs per patient in hospital		9.22±5.81	
Number of pDDIs per patient after discharge		2.38±2.44	

### Epidemiology and severity of pDDIs (pCDIs+pHDIs)

**Figure 1:**
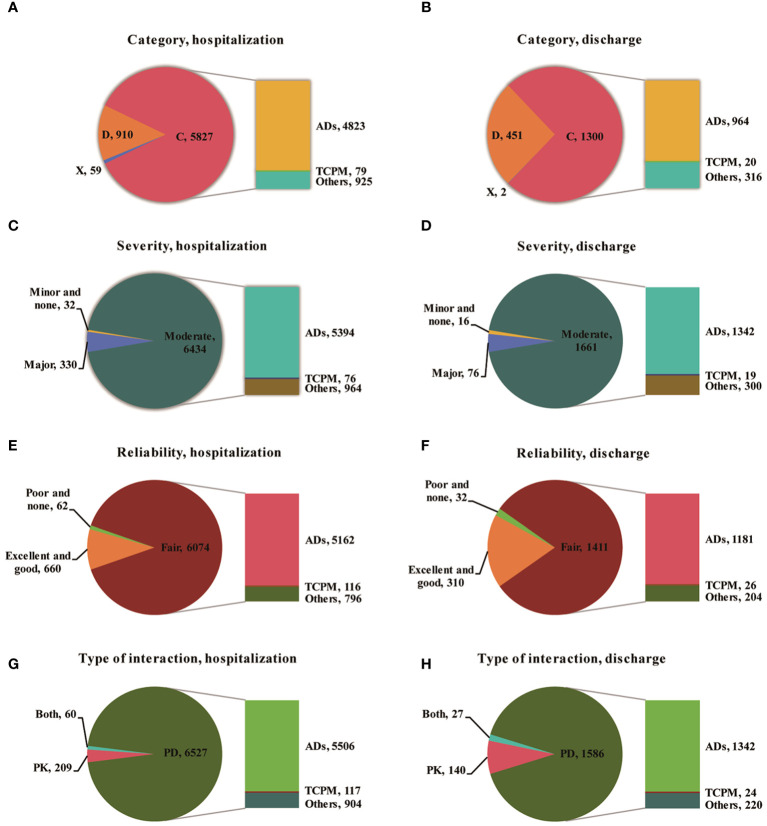
The characteristics distribution including catepgory, severity, reliability, type of interaction of pDDIs during hospitalization and after discharge (**A**,Category of pDDIs during hospitalization; **B**, Category of pDDIs after discharge; **C**, Severity of pDDIs during hospitalization; **D**, Severity of pDDIs after discharge; **E**, Reliabilityof pDDIs during hospitalization; **F**, Reliabilityof pDDIs after discharge; **G**, Type of interaction during hospitalization; **H**, Type of interaction Type of interaction; ADs, Antidiabetic Drugs Involved; TCPM, Traditional Chinese Patent Medicine; PK, Pharmacokinetics; PD, Pharmacodynamics; Both, PK AND PD).

A total of 6796 pDDIs (6674 pCDIs and 122 pHDIs) during hospitalization and 1753 pDDIs (1727 pCDIs and 26 pHDIs) after discharge were identified in this study. On average, there were 9.22 pDDIs per patient during hospitalization and 2.38 after discharge. Among the 6796 pDDIs detected during hospitalization, 59 (0.87%) were classified as X risk rating, 910 (13.39%) as D risk rating, and 5827 (85.74%) as C risk rating. Among the 1753 pDDIs detected after discharge, 2 (0.11%) were classified as X risk rating, 451 (25.73%) as D risk rating, and 1300 (74.16%) as C risk rating (**Figure 1**). Even more to the point, one patient may have multiple pCDIs or pHDIs, with the number of interactions ranging from 1 to 55 interactions per person during hospitalization and from 1 to 16 after discharge. Therefore, pCDIs and pHDIs with the same or different severity levels were manifested in many medical orders.

**Figure 2:**
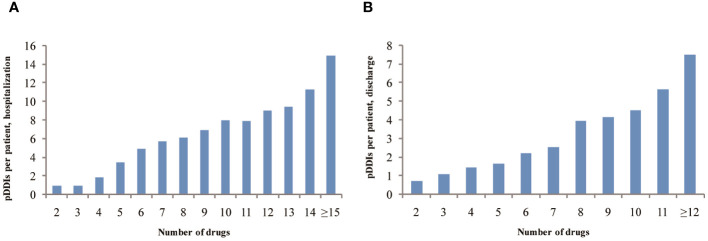
The prevalence of pDDIs as a function of number of dispensed drugs among 737 T2DM hospitalized patients during hospitalization **(A)** or after discharge **(B)**.

More than 80% of pDDIs were found to be associated with hypoglycemia. The majority of pCDIs and pHDIs were rated as C risk ratings with moderate interaction severity, both during hospitalization and after discharge. The most serious pCDIs and pHDIs (type X) were found in 59 patients (0.87%), involving 54 pCDIs and 5 pHDIs during hospitalization and 2 (0.11%) after discharge. According to the reliability rating, 89.38% of the interactions during hospitalization and 80.49% after discharge were classified as fair. The predominant underlying mechanisms for pDDIs were pharmacodynamics (PD), accounting for 96.04% during hospitalization and 90.47% after discharge. Pharmacokinetics (PK), including decreased drug absorption, enhanced drug concentration, and inhibition of cytochrome P450 enzymes, accounted for 3.08% during hospitalization and 7.99% after discharge. The prevalence of pDDIs among 737 T2DM hospitalized patients increased with the number of dispensed drugs both during hospitalization and after discharge (**Figure 2**).

**Figure 3:**
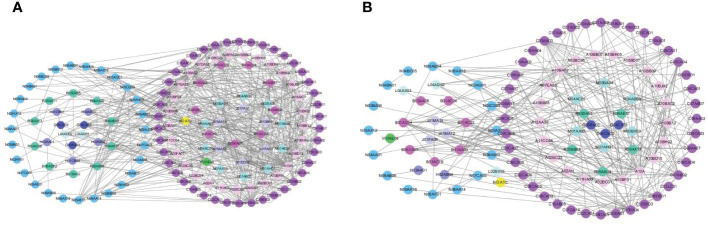
The chemical drug-drug association networks during hospitalization **(A)** or after discharge **(B)**.

The networks were constructed based on pCDIs during hospitalization and after discharge (**Figure 3**). The nodes represented drugs, and the links between pairs of drugs represented pCDIs between them. According to the anatomical therapeutic chemical (ATC) classification system, 145 different medications were administered during hospitalization and 108 different medications after discharge, which were then clustered into 11 main groups. Each node represented one drug and was colored according to the first-level ATC classification. Majority drugs interacted with only a limited number of other drugs, whereas only a minority of drugs interacted with numerous other drugs. The majority of pCDIs were associated with cardiovascular system drugs, both during hospitalization and after discharge, followed by alimentary tract, metabolism, and nervous system drugs.

A comprehensive list of potential adverse outcomes and the type of interaction for all drug interactions belonging to X rating during hospitalization was presented in **Table 2**. The most prevalent interacting pair of pDDIs within this category was chlorphenamine-potassium chloride, accounting for 30.51% of the total interactions. The most frequent potential adverse outcome during hospitalization was the increased ulcerative effect of potassium chloride, accounting for 76.27% of the potential adverse outcomes in the X category. The high risks of bleeding and gastrointestinal toxicity were also discovered in T2DM patients. Danshen-warfarin was the only drug pair of pHDIs among the X rating with the risk of increased bleeding effect of warfarin.

**Table 2:** PDDIs belonging to X rating with potential adverse outcomes and type of interaction during hospitalization (PK, Pharmacokinetics; PD, Pharmacodynamics).

Drug combinations	Potential adverse outcomes	Type of interaction	N (%)
Chlorphenamine-potassium chloride	Increased the ulcerative effect of potassium chloride	PD	18(30.51)
Ketotifen-potassium chloride	Increased the ulcerative effect of potassium chloride	PD	9(15.25)
Flupentixol-potassium chloride	Increased the ulcerative effect of potassium chloride	PD	6(10.17)
Melitracen-potassium chloride	Increased the ulcerative effect of potassium chloride	PD	6(10.17)
Danshen-warfarin	Increased the bleeding effect of warfarin	PD	5(8.47)
Cetirizine-potassium chloride	Increased the ulcerative effect of potassium chloride	PD	5(8.47)
Pantoprazole-cefuroxime	Decreased the absorption of cefuroxime	PK	1(1.69)
Ebastine-potassium chloride	Increased the ulcerative effect of potassium chloride	PD	1(1.69)
Ibuprofen-celecoxib	Increased the adverse/toxic effect of celecoxib, especially gastrointestinal toxicity	PD	1(1.69)
Indometacin-celecoxib	Increased the adverse/toxic effect of celecoxib, especially gastrointestinal toxicity	PD	1(1.69)
Dipyrone-indometacin	Increased the adverse/toxic effect of indometacin, especially gastrointestinal toxicity	PD	1(1.69)
Hyoscine-potassium chloride	Increased the ulcerative effect of potassium chloride	PD	1(1.69)
Aminophylline-doxofylline	Increased the adverse/toxic effect of doxofylline	PD	1(1.69)
Omeprazole-cefuroxime	Decreased the absorption of cefuroxime	PK	1(1.69)
Clozapine-potassium chloride	Increased the ulcerative effect of potassium chloride	PD	1(1.69)
Perphenazine-potassium chloride	Increased the ulcerative effect of potassium chloride	PD	1(1.69)

The common (top 20) pDDIs in category D with potential adverse outcomes, severity, reliability and type of interaction during hospitalization, and the pDDIs frequency of the drug combinations after discharge were demonstrated in **Table 3**. The majority of pDDIs in category D were associated with antidiabetic drugs with the potential risk of hypoglycemia both during the hospital stay and after discharge. Licorice-aspirin, ginseng-aspirin and licorice-clopidogrel were the three types of pHDIs pairs among the top 20 pDDIs of category D with the mechanism of enhanced the adverse/toxic effect of aspirin or clopidogrel, especially the risk of bleeding. The most frequent pDDIs in D rating involving non-antidiabetic drugs after discharge were between omeprazole and clopidogrel. Apparently, the pDDIs related to antiplatelet drugs such as aspirin and clopidogrel made up a significant proportion of D rating non-hypoglycemic related pDDIs.

**Table 3:** Common (top 20) pDDIs belonging to D category with potential adverse outcomes, severity, reliability and type of interaction during hospitalization, and the pDDIs frequency of the drug combinations after discharge.

Drug combinations	Potential adverse outcomes	Severity	Reliability	Type of interaction	N (%)	
					during hospitalization	after discharge
Acarbose-insulin	Increased the risk of hypoglycemia	Moderate	Fair	PD	556 (61.10)	317 (70.29)
Dapagliflozin-insulin	Increased the risk of hypoglycemia	Major	Fair	PD	98 (10.77)	48 (10.64)
Vildagliptin-insulin	Increased the risk of hypoglycemia	Major	Fair	PD	44 (4.84)	23 (5.10)
Acarbose-glimepiride	Increased the risk of hypoglycemia	Moderate	Fair	PD	16 (1.76)	16 (3.55)
Omeprazole-clopidogrel	Diminished the antiplatelet effect of clopidogrel and the serum concentrations of its active metabolites	Major	Fair	PK	14 (1.54)	7 (1.55)
Chlorpheniramine-opium tincture	Enhanced the CNS depressant effect	Major	Fair	PD	12 (1.32)	0 (0.00)
Licorice-aspirin	Increased the adverse/toxic effect of aspirin, especially bleeding effect	Major	Fair	PD	11 (1.21)	1 (0.22)
Repaglinide-clopidogrel	Increased the serum concentrations of repaglinide	Major	Good	PK	10 (1.10)	7 (1.55)
Spirolactone-potassium chloride	Increased the hyperkalemic effect	Major	Fair	PD	10 (1.10)	0 (0.00)
ketotifen-opium tincture	Enhanced the CNS depressant effect	Major	Fair	PD	9 (0.99)	0 (0.00)
Ferrous succinate-thioctic acid	Decreased the absorption of ferrous succinate	Moderate	Fair	PK	8 (0.88)	0 (0.00)
Aspirin-celecoxib	Increased the adverse/toxic effect of celecoxib	Major	Good	PD	6 (0.66)	0 (0.00)
Liraglutide-insulin	Increased the risk of hypoglycemia	Moderate	Fair	PD	6 (0.66)	3 (0.67)
Acarbose-gliclazide	Increased the risk of hypoglycemia	Moderate	Fair	PD	6 (0.66)	1 (0.22)
Ginseng-aspirin	Increased the adverse/toxic effect of aspirin, especially bleeding effect	Major	Fair	PD	6 (0.66)	1 (0.22)
Indometacin-aspirin	Increased the adverse/toxic effect of aspirin, especially bleeding effect	Moderate	Good	PK AND PD	5 (0.55)	0 (0.00)
Licorice-clopidogrel	Increased the adverse/toxic effect of clopidogrel, especially bleeding effect	Major	Fair	PD	5 (0.55)	1 (0.22)
Dapagliflozin-glimepiride	Increased the risk of hypoglycemia	Major	Fair	PD	4 (0.44)	2 (0.44)
Sitagliptin-insulin	Increased the risk of hypoglycemia	Major	Fair	PD	4 (0.44)	1 (0.22)
Clopidogrel-heparin	Increased the anticoagulant effect	Moderate	Fair	PD	3 (0.33)	0 (0.00)

### Factors associated with pDDIs (pCDIs+pHDIs)

The univariable logistic regression analysis revealed significant associations between the occurrence of over 4 pDDIs during hospitalization and the following factors: complications of T2DM [OR (95% confidence interval (CI): 1.89 (1.20–2.97), p<0.05], number of coexisted diseases [OR (95% CI): 1.70 (1.10–2.63), p<0.05], high BMI (≥30) [OR (95% CI): 6.00 (1.17–30.77), p<0.05], and number of administered medications [OR (95% CI): 6.62 (4.11–10.65) and 48.63 (16.84–140.42), p<0.05] (**Table 4**). There were no significant correlations between gender, age, length of hospitalization, history of T2DM, alcohol use history, tobacco use history, family history of T2DM and pDDIs during hospitalization (p>0.05). Moreover, age [OR (95% CI): 2.18 (1.22–3.90) and 2.36 (1.29–4.30), p<0.05], length of hospitalization [OR (95% CI): 1.64 (1.08–2.50), p<0.05], length of T2DM history [OR (95% CI): 2.04 (1.21–3.44), p<0.05], complications of T2DM [OR (95% CI): 2.90 (1.87–4.50), p<0.05], and the number of discharge medications [OR (95% CI): 5.01 (3.04–8.27), p<0.05] were found to be independently correlated with the occurrence of pDDIs after discharge (**Table 5**). While, gender, alcohol use history, tobacco use history, family history of T2DM and the number of coexisted diseases showed no correlation with the occurrence of pDDIs after discharge (p>0.05).

**Table 4:** Univariate and multivariate analyses of factors associated to pDDIs during hospitalization.

Variables	PDDIs (>4)		Univariate		Multivariate	
	Yes n	No n	OR (95% CI)	p-Value	OR (95% CI)	p-Value
Gender						
Male	345	66	1			
Female	282	44	1.23 (0.81-1.85)	0.333		
Age (years)						
18-39	65	16	1			
40-64	305	49	1.53 (0.82-2.86)	0.181		
≥65	257	45	1.41 (0.75-2.64)	0.291		
BMI						
Underweight (<18.5 kg/m^2^)	10	3	1		1	
Normal (18.5-24.99 kg/m^2^)	259	69	1.13 (0.30-4.20)	0.860	0.19 (0.031-1.20)	0.078
Preobese (25-29.99 kg/m^2^)	278	34	2.45 (0.64-9.35)	0.189	0.20 (0.067-0.60)	0.004
Obese (≥30 kg/m^2^)	80	4	6.00 (1.17-30.77)	0.032	0.47 (0.16-1.45)	0.192
Length of hospitalization, (day)						
1-10	330	68	1		1	
>10	297	42	1.46 (0.96-2.21)	0.076	1.09 (0.67-1.75)	0.735
History of T2DM, (years)						
0-10	445	84	1			
>10	182	26	1.32 (0.82-2.12)	0.248		
Alcohol use history						
No	588	102	1			
Yes	39	8	0.85 (0.38-1.86)	0.677		
Tobacco use history						
No	561	95	1			
Yes	66	15	0.74 (0.41-1.36)	0.338		
Family history of T2DM						
No	445	80	1			
Yes	182	30	1.09 (0.69-1.72)	0.708		
Complications of T2DM						
No	116	33	1		1	
Yes	511	77	1.89 (1.20-2.97)	0.006	0.99 (0.57-1.73)	0.995
The number of coexisted diseases						
0-2	144	37	1		1	
≥3	483	73	1.70 (1.10-2.63)	0.017	0.68 (0.40-1.15)	0.150
Administered medications during hospitalization						
2-7	54	52	1		1	
8-13	371	54	6.62 (4.11-10.65)	0.000	7.65 (4.47-13.11)	0.000
>13	202	4	48.63 (16.84-140.42)	0.000	55.05 (17.97-168.67)	0.000

**Table 5:** Univariate and multivariate analysis of factors associated to pDDIs after discharge.

Variables	PDDIs		Univariate analysis		Multivariate analysis	
	Yes n	No n	OR (95% CI)	p-Value	OR (95% CI)	p-Value
Gender						
Male	344	67	1			
Female	284	42	1.32 (0.87-2.00)	0.195		
Age (years)						
18-39	60	21	1		1	
40-64	305	49	2.18 (1.22-3.90)	0.009	1.30 (0.62-2.74)	0.483
≥65	263	39	2.36 (1.29-4.30)	0.005	1.56 (0.93-2.61)	0.091
BMI						
Underweight (<18.5 kg/m^2^)	9	4	1		1	
Normal (18.5-24.99 kg/m^2^)	279	49	2.53 (0.75-8.54)	0.125	0.32 (0.07-1.34)	0.118
Preobese (25-29.99 kg/m^2^)	266	46	2.57 (0.76-8.69)	0.129	0.84 (0.37-1.84)	0.662
Obese (≥30 kg/m^2^)	74	10	3.29 (0.852-12.69)	0.084	0.84 (0.38-1.84)	0.666
Length of hospitalization, (day)						
1-10	328	70	1		1	
>10	300	39	1.64 (1.08-2.50)	0.021	1.50 (0.96-2.35)	0.073
History of T2DM, (years)						
0-10	439	90	1		1	
>10	189	19	2.04 (1.21-3.44)	0.008	0.64 (0.36-1.14)	0.130
Alcohol use history						
No	590	100	1			
Yes	38	9	0.72 (0.34-1.52)	0.386		
Tobacco use history						
No	561	95	1			
Yes	67	14	0.81 (0.44-1.50)	0.503		
Family history of T2DM						
No	446	79	1			
Yes	182	30	1.07 (0.68-1.69)	0.756		
Complications of T2DM						
No	108	41	1		1	
Yes	520	68	2.90 (1.87-4.50)	0.000	2.10 (1.26-3.52)	0.005
The number of coexisted diseases						
0-2	152	29	1			
≥3	476	80	1.13 (0.71-1.80)	0.591		
Discharge medications						
0-5	286	88	1		1	
≥6	342	21	5.01 (3.04-8.27)	0.000	4.16 (2.44-7.11)	0.000

Multivariate analysis indicated that T2DM patients who received 8–13 medications or more than 13 medications during hospitalization had 7.65 and 55.05 times higher odds of developing pDDIs compared to those who received 2–7 drugs [OR (95% CI): 7.65 (4.47–13.11); OR (95% CI): 55.05 (17.97–168.67), p < 0.05], respectively. The presence of pDDIs after discharge was associated with the complications of T2DM and the number of discharge medications [OR (95% CI): 2.10 (1.26–3.52); OR (95% CI): 4.16 (2.44–7.11), p<0.05,respectively]. The number of administered medications was the most strongly associated variable, both during hospitalization and after discharge.

## Discussion

A total of 6796 pDDIs (6674 pCDIs and 122 pHDIs) were identified during hospitalization, while 1753 (1727 and 26) were observed after discharge among T2DM inpatients. The prevalence of pDDIs was 99.73% during their hospital stay and 85.21% after discharge, which were significantly higher than those reported in previous similar studies (14, 15). A retrospective study conducted by Ivana Samardzic et al. revealed that 80.9% of patients with diabetes mellitus were exposed to at least one pDDI, but only category C interactions were detected by the Lexi-Interact software (14). While Ilona Ikäheimo et al. found that clinically relevant DDIs were presented in 44.5% of patients aged ≥65 years with T2DM in Finnish home-dwelling primary care (15). However, direct comparison between these studies was challenging due to discrepancies in study design, prescribed medications, pDDIs checkers used, criteria for identifying pDDIs and other factors (16). The majority of the identified pDDIs were associated with antidiabetic drugs both during hospitalization and after discharge. Approximately 31.90% of the total identified pDDIs during hospitalization involved interactions between hypoglycemic medications, while this percentage increased to 58.47% after discharge. The higher number of pDDIs observed among inpatients compared to those after discharge demonstrated an increased risk of DDIs during hospitalization. Nevertheless, more importantly, not all pDDIs would actually occur, as they are only considered as potential hazards. Additionally, some patients may encounter new diseases or complications after discharge from the hospital and may take additional medications that could initiate new pDDIs. Therefore, minimizing the risk of pDDIs is of the essence in T2DM patients.

Despite most pDDIs reported in our study had a C risk rating with moderate severity, more attention was warranted regarding the higher severity classifications of pDDIs. Among T2DM inpatients, the most frequently identified pCDIs in category X were antihistamines and potassium chloride, resulting in adverse reactions and an increased risk of ulcerative effects. Interactions with nonsteroidal anti-inflammatory drugs (NSAIDs) such as ibuprofen, celecoxib, indomethacin, and celecoxib were detected in our study population under category X. Some studies have suggested that regardless of the chemical class, all NSAIDs could produce augmented dose-dependent analgesia and gastromucosal injury with associated mucosal barrier dysfunction after extended use (2 weeks) (17). The concurrent use of NSAIDs, which represents therapeutic duplication (18), should be circumvented due to the increased toxic effects, especially gastrointestinal toxicity. Unfortunately, we have not collected any information on whether patients experienced gastrointestinal consequences as a result of this combination. The PD interaction between omeprazole and clopidogrel was the most frequently observed pDDI under category D among non-antidiabetic drugs. It was identified in 14 patients (1.54%) during hospitalization or in 7 patients (1.55%) after discharge, with the risk of therapy failure and a loss of clopidogrel’s protective cardiovascular benefits. Omeprazole could reduce the antithrombotic efficacy of clopidogrel by inhibiting the activity CYP2C19, resulting in lower serum concentrations of the active metabolite (19, 20). Studies in a multi-ethnic Asian population had shown that the concomitant use of omeprazole with clopidogrel was associated with an increased risk of myocardial infarction, but not with mortality or stroke (21). Thus, it was strongly recommended to replace omeprazole with rabeprazole or pantoprazole for patients using clopidogrel, as rabeprazole and pantoprazole had low CYP2C19 inhibitory potential (22, 23).

In our study, the proportion of T2DM inpatients who concurrently used Chinese patent drugs decreased from 7.46% during hospitalization to 2.44% after discharge. Among the pHDIs in X category retrieved from hospitalized patients, the combination of danshen and warfarin was found to be associated with an increased risk of bleeding effects (24). This was attributed to the enhanced bioavailability of both R- and S-warfarin when used concomitantly with danshen, leading to an exaggerated anticoagulant response (25). Previous reports had indicated that patients receiving warfarin therapy should avoid consuming herbal products, including danshen, ginseng, ginkgo, and dong quai because of their high risk of bleeding (26, 27). The major tanshinones in danshen had been reported to elevate the steady-state plasma concentration of warfarin by 23% through inhibition of warfarin hydroxylation (28, 29). Nonetheless, safe coadministration might be achievable with close monitoring and adjustment of the warfarin dosage. Specific herbs with antiplatelet activity, such as ginkgo, ginseng, and licorice, were detected under category D among non-antidiabetic drugs that could interact with clopidogrel or aspirin, potentially increasing the risk of bleeding. Although laboratory studies had indicated the potential for pHDIs between these herbs and aspirin or clopidogrel, there may not necessarily be a significant clinical correlation (30–32). However, until more evidence regarding their safety is available, it is advisable to avoid co-administration of these herbs with aspirin or clopidogrel.

This study showed a statistically significant correlation between the number of medications and the risk of pDDIs, both during hospital stays and after discharge. Previous studies had also found that the risk of DDIs increased with the number of medications, especially when they were from diverse pharmacological categories. This could be expected as numerous drugs had overlapping elimination pathways and non-selective mechanisms of action. Other factors associated with pDDIs after discharge included complications of T2DM. T2DM could induce various complications, resulting in an increased number of prescribed medications and consequently leading to an elevated risk of pDDIs.

However, there were some limitations in this study. First of all, only one pDDIs checker was utilized to evaluate the prevalence of pDDIs. Employing multiple pDDIs checkers could potentially improve the accuracy of identifying these interactions. Secondly, this study had a retrospective design and was conducted at a single center. It could not track the medications that patients self-administered after discharged or investigate the relationship between clinical outcomes and pDDIs. Although future multicenter and prospective studies in clinical settings were still needed to address all these limitations, this study was the first to elucidate the issues concerning pCDIs and pHDIs in T2DM patients, both during hospitalization and after discharge.

## Conclusion

In this study, T2DM patients were exposed to a significant number of pDDIs, including pCDIs and pHDIs, both during hospitalization and after discharge. The concurrent use of multiple drugs was identified as the most significant risk factor for pDDIs. The utilization of drug interaction software, electronic warning systems, and prescription pre-review systems, along with the collaboration of clinical pharmacists, has the potential to dramatically reduce the potentially harmful drug combinations and contribute to enhancing patient safety. The development of universally applicable clinical guidelines for pDDIs may efficiently recognize pDDIs and provide further evidence to support clinically rational drug use.

## Data availability statement

The raw data supporting the conclusions of this article will be made available by the authors, without undue reservation.

## Ethics statement

The studies involving humans were approved by Jinshan Hospital of Fudan University Ethics Committee. The studies were conducted in accordance with the local legislation and institutional requirements. Written informed consent for participation was not required from the participants or the participants’ legal guardians/next of kin in accordance with the national legislation and institutional requirements. Written informed consent was obtained from the individual(s) for the publication of any potentially identifiable images or data included in this article.

## Author contributions

WR: Resources, Data curation, Writing – original draft, Investigation, Funding acquisition. YL: Investigation, Formal analysis, Writing – review & editing, Software. HJ: Writing – review & editing, Investigation. XL: Formal analysis, Writing – review & editing, Supervision. NZ: Writing – review & editing, Supervision.
